# Alternative dosing regimens for atezolizumab: an example of model-informed drug development in the postmarketing setting

**DOI:** 10.1007/s00280-019-03954-8

**Published:** 2019-09-21

**Authors:** Kari M. Morrissey, Mathilde Marchand, Hina Patel, Rong Zhang, Benjamin Wu, H. Phyllis Chan, Almut Mecke, Sandhya Girish, Jin Y. Jin, Helen R. Winter, René Bruno

**Affiliations:** 1 Clinical Pharmacology, Genentech, Inc, 1 DNA Way, South San Francisco, CA 94080 USA; 2 Certara Strategic Consulting, Certara, 54 Rue de Londres, 75009 Paris, France; 3 Safety Science Oncology, Genentech Inc, 1 DNA Way, South San Francisco, CA 94080 USA; 4 Biostatistics, F. Hoffmann-La Roche, Ltd, Hochstrasse 16, 4053 Basel, Switzerland; 5 Clinical Pharmacology, Genentech/Roche, 84 Chemin des Grives, 13013 Marseille, France; 6 Present Address: Seattle Genetics, 21717 30th Dr SE, Bothell, WA 98021 USA

**Keywords:** Atezolizumab, PD-L1, Population pharmacokinetics, Exposure–response

## Abstract

**Purpose:**

To determine the exposure–response (ER) relationships between atezolizumab exposure and efficacy or safety in patients with advanced non-small cell lung cancer (NSCLC) or urothelial carcinoma (UC) and to identify alternative dosing regimens.

**Methods:**

ER analyses were conducted using pooled NSCLC and UC data from phase 1 and 3 studies (PCD4989g, OAK, IMvigor211; ClinicalTrials.gov IDs, NCT01375842, NCT02008227, and NCT02302807, respectively). Objective response rate, overall survival, and adverse events were evaluated vs pharmacokinetic (PK) metrics. Population PK-simulated exposures for regimens of 840 mg every 2 weeks (q2w) and 1680 mg every 4 weeks (q4w) were compared with the approved regimen of 1200 mg every 3 weeks (q3w) and the maximum assessed dose (MAD; 20 mg/kg q3w). Phase 3 IMpassion130 (NCT02425891) data were used to validate the PK simulations for 840 mg q2w. Observed safety data were evaluated by exposure and body weight subgroups.

**Results:**

No significant ER relationships were observed for safety or efficacy. Predicted exposures for 840 mg q2w and 1680 mg q4w were comparable to 1200 mg q3w and the MAD and consistent with observed PK data from IMpassion130. Observed safety was similar between patients with a *C*_max_ above and below the predicted *C*_max_ for 1680 mg q4w and between patients in the lowest and upper 3 body weight quartiles.

**Conclusion:**

Atezolizumab regimens of 840 mg q2w and 1680 mg q4w are expected to have comparable efficacy and safety as the approved regimen of 1200 mg q3w, supporting their interchangeable use and offering patients greater flexibility.

**Electronic supplementary material:**

The online version of this article (10.1007/s00280-019-03954-8) contains supplementary material, which is available to authorized users.

## Introduction

Immune checkpoint inhibition targeting programmed death-ligand 1 (PD-L1) or programmed death-1 (PD-1) has become an important approach in the treatment of multiple human cancers, as PD-L1 expression on tumor cells and tumor-infiltrating immune cells can inhibit anticancer immune responses [1]. Atezolizumab, a humanized, engineered monoclonal immunoglobulin (Ig) G1 antibody, selectively targets PD-L1 to block interactions with its receptors to promote T-cell activation and reinvigorate and enhance anticancer activity, while leaving the interaction between PD-L2 and PD-1 intact [1–3]. Atezolizumab is approved to treat certain types of locally advanced or metastatic non-small cell lung cancer (NSCLC) and urothelial carcinoma (UC) in the United States, Europe, and elsewhere, as well as locally advanced or metastatic triple-negative breast cancer (TNBC) and extensive-stage small-cell lung cancer (SCLC) in the United States [4, 5].

The first-in-human phase 1 dose-escalation and dose-expansion study PCD4989g evaluated atezolizumab monotherapy in patients with locally advanced or metastatic cancers using intravenous (IV) infusions of 0.01 to 20 mg/kg doses every 3 weeks (q3w) as well as a 1200 mg flat-dose equivalent of 15 mg/kg q3w [3]; no dose-limiting toxicities were observed, and no maximum tolerated dose was identified [3]. The pharmacokinetics (PK) of atezolizumab observed in this and subsequent clinical studies were consistent with the expected profile of an IgG1 antibody in humans, with a terminal half-life of 27 days [4, 6]. Atezolizumab demonstrated linear PK over the dose range of 1–20 mg/kg IV q3w, including the 1200 mg dose. The clearance was 0.2 L/day, and the volume of distribution at steady state (achieved after 6–9 weeks of treatment) was 6.9 L. Clearance was found to decrease with time (mean maximal reduction from baseline of approximately 17%), but this decrease did not appear to be clinically relevant [4]. Selection of the 1200 mg q3w dosing regimen for further study was informed by nonclinical studies identifying a target serum exposure for atezolizumab [7] [minimum (trough) serum concentration (*C*_min_) of 6 μg/mL] and available PK and exposure–response (ER) data from several clinical studies, including PCD4989g and the pivotal phase 2 study IMvigor210 in locally advanced or metastatic UC, indicating that this target was achieved at 1200 mg q3w for > 95% of patients [6].

The UC and NSCLC atezolizumab monotherapy indications, as well as the NSCLC and SCLC atezolizumab combination therapy indications, were first approved for IV infusions of 1200 mg q3w; the TNBC atezolizumab plus nab-paclitaxel combination indication was US Food and Drug Administration (FDA) approved at an atezolizumab dose of 840 mg every 2 weeks (q2w). Identification of alternative dosing regimens that can be used interchangeably would offer patients greater convenience in their cancer treatment, particularly for combination regimens with diverse dosing requirements. The goals of this study were to determine the atezolizumab ER relationship for efficacy and safety and to apply this knowledge, along with population PK (popPK) simulations and the known safety profile of atezolizumab, to identify alternative dosing regimens. This “exposure-matching” approach to identify new dosing regimens is consistent with the model-informed drug development pilot programs endorsed by the FDA [8, 9], and these data have led to the expansion of the dosing regimens for several atezolizumab indications [4]. Accordingly, in May 2019, the 840 mg q2w and 1680 mg q4w dosing regimens were added to the FDA-approved UC, NSCLC and SCLC indications where atezolizumab is used as a single agent.

## Materials and methods

### Studies

Data from atezolizumab monotherapy studies, including the phase 1 study PCD4989g (NSCLC and UC cohorts only; ClinicalTrials.gov ID, NCT01375842) [3, 10–12] and phase 3 studies OAK (NSCLC, GO28915; ClinicalTrials.gov ID, NCT02008227) [13] and IMvigor211 (UC, GO29294; ClinicalTrials.gov ID, NCT02302807) [14] (Table S1), were used in the ER analyses based on clinical data cut-off dates of December 2, 2014; July 7, 2016; and March 13, 2017, respectively. OAK and IMvigor211, randomized studies of atezolizumab monotherapy vs chemotherapy, were used in tumor growth inhibition (TGI) modeling analyses. PK simulations were performed based on the popPK model [6] developed with Phase I data (*n* = 472) from PCD4989g and JO28944, the phase 1 study conducted in Japanese patients [15] and validated with data from over nine studies, including phase 3 studies OAK, IMvigor211, and IMpassion130 (WO29522; ClinicalTrials.gov ID, NCT02425891) [16]. Safety data for select patient subgroups from PCD4989g (using a data cut-off date of March 31, 2016) and OAK were summarized. The protocols for studies from which data were analyzed were approved by institutional review boards and/or independent ethics committees at each site. All patients provided written informed consent.

### ER evaluations: measures, endpoints, and analyses

ER analyses were performed to inform any relationships between PK metrics and ORR, OS, grade 3–5 adverse events (AEs), and AE of special interest (AESI) endpoints evaluated in previous clinical studies based on cycle 1 data to minimize potential bias due to both confounding with baseline prognostic factors [17, 18] and time-dependent variation in clearance that has been observed for atezolizumab and other checkpoint inhibitors [4, 8, 19–22]. These analyses were conducted using pooled data from atezolizumab-treated patients with NSCLC or UC (from PCD4989g, OAK, and IMvigor211) for whom exposure data were available, except as noted below for overall survival (OS). Exploratory ER analyses were performed using cycle 1 maximum serum concentration (*C*_max_), *C*_min_, and area under the concentration–time curve (AUC; time 0–21 days), as recommended [21] to minimize the effect of response-dependent time-varying clearance observed previously for anti-PD-1 and anti-PD-L1 agents [20]. AUC (time 0–21 days), *C*_max_, and *C*_min_ were derived at cycle 1 based on individual PK parameters estimated using cycle 1 data only and the previously developed popPK model [6]. The efficacy endpoints evaluated were investigator-assessed confirmed Response Evaluation Criteria in Solid Tumors version 1.1 (RECIST 1.1) objective response rate (ORR; secondary endpoint in all studies) and OS (primary endpoint in OAK and IMvigor211). ORR analyses used data from atezolizumab-treated patients with NSCLC or UC in PCD4989g, OAK (first 850 randomized patients), and IMvigor211, whereas OS analyses used data from OAK (first 850 randomized patients) and IMvigor211 only. The safety endpoints evaluated included AEs of grades 3–5 per National Cancer Institute Common Terminology Criteria for Adverse Events version 4 and Medical Dictionary for Regulatory Activities version 20.1 (primary endpoint in PCD4989g, also evaluated in OAK and IMvigor211) and AESIs (evaluated in all studies). AESIs, conditions suggestive of autoimmune disorder, were defined previously [11].

ORR and AEs were evaluated as binary endpoints (yes/no) and studied vs exposure as a continuous variable using logistic regression. The Wald test *P* value was reported for each logistic regression, along with proportions/frequencies and their 95% CIs computed for quartiles of exposure. For OS data, to mitigate confounding factors between patients’ baseline information and atezolizumab clearance and exposure, TGI-OS modeling [23, 24] was performed. To be evaluable in this analysis (TGI evaluable), patients needed to have ≥ 1 posttreatment sum of longest diameters (SLD) assessment. The impact of individual baseline prognostic factors and TGI metrics (estimated tumor shrinkage and tumor growth rates in a biexponential longitudinal model of the SLD of the target lesions per RECIST 1.1) on OS were explored using Kaplan–Meier and Cox regression analyses, and a parametric multivariate regression TGI-OS model was built. The final TGI-OS model was validated by simulation in its ability to describe OS distributions and hazard ratios (HRs) compared with a control in different subgroups (notably by exposure quartiles). For the HR simulations, TGI metric estimates and baseline covariates for control patients were taken from previous analyses [24, 25]. Exposure metrics were tested on the final multivariate model after adjustment for confounding with prognostic factors. A “tumor type” factor could be incorporated in the model if appropriate.

### PopPK simulations

To simulate PK parameters of varying regimens of atezolizumab [840 mg q2w, 1200 mg q3w, 1680 mg every 4 weeks (q4w), and 20 mg/kg q3w], Monte Carlo simulations were performed using the popPK model of atezolizumab, including covariate effects, previously developed using PCD4989g data [6] to obtain virtual individual PK profiles at cycle 1 and steady state. In the popPK model used for the PK simulations, bodyweight, albumin, tumor burden, treatment-emergent antidrug antibody (ADA) status, and gender were found to have a statistically significant impact on atezolizumab PK [6]. A single replicate of 500 patients was simulated for each regimen. A seed number was provided in the control stream to ensure reproducibility of the simulations. Random effects were sampled from the previously estimated distribution, and the residual error was not taken into account for individual predictions. Virtual patients per dosing regimen were assumed to have a 1:1 male:female ratio (males weighing 85 kg and females weighing 64 kg, median body weight in the phase 1 database used to develop the popPK model). Other covariates affecting atezolizumab PK parameters were set to the median or most frequent category for the categorical covariates: albumin level of 40 g/L, baseline tumor size of 63 mm, and negative for ADAs. Four dosing regimens were simulated: 1200 mg q3w, 20 mg/kg q3w (i.e., 1700 mg for males and 1280 mg for females), 840 mg q2w, and 1680 mg q4w. To assess the impact of body weight on exposure after the fixed-dose regimen, 500 virtual patients per quartile of body weight with median albumin level, baseline tumor size, and negative for ADAs were assigned a dose of 840 mg q2w or 1680 mg q4w. The distribution of body weight in the phase 1 population of patients was divided by quartiles as follows: 36.5–63.7, 63.7–77.0, 77.0–90.9, and 90.9–168.0 kg. The 500 individual body weights were sampled in each quartile assuming a truncated normal distribution. To maintain the correlation between sex and body weight, the proportion of females was set to 80% in the first quartile, 50% in the second quartile, 25% in the third quartile, and 10% in the last quartile, as observed in the phase 1 database used to develop the popPK model.

Atezolizumab exposure metrics (cycle 1: AUC [calculated using the trapezoidal method; time 0–21 days], *C*_max_, and *C*_min_; steady state: AUC [dose/clearance], *C*_max_, and *C*_min_) were derived from the simulated individual PK profiles and summarized across individuals for each dosing regimen. To compare several dosing regimens involving different dosing intervals (every 2, 3, or 4 weeks), steady-state weekly AUC data were also derived.

### Validation of 840 mg q2w exposure data from popPK predictions

A prediction-corrected visual predictive check (pcVPC) was performed based on the prior phase 1 popPK model (external evaluation). The phase 1 popPK model was used to derive the individual PK parameter estimates based on atezolizumab observed concentration–time profiles in IMpassion130. PK data for atezolizumab-treated patients in IMpassion130 were simulated (1000 replicates) using actual dosing and patient covariates (body weight, sex, ADA status, albumin level, and tumor burden) and the phase 1 popPK model. Observed atezolizumab peak (*C*_max_) and trough (*C*_min_) concentrations in IMpassion130 were compared with corresponding predictive distributions.

### Additional safety analyses

AE frequencies were summarized for subgroups of patients: (1) from PCD4989g who received atezolizumab 20 mg/kg q3w based on *C*_max_ values in relation to predicted *C*_max_ for the 1680 mg q4w regimen, (2) from PCD4989g based on the atezolizumab dose received, and (3) from PCD4989g and OAK based on body weight quartiles (lowest quartile vs quartiles 2–4). In the PCD4989g study, adverse events were collected until 90 days following the last administration of study treatment or until initiation of the subsequent anticancer therapy, whichever occurred first (Figure S6, footnote 1). In these analyses, whether or not AESIs required the use of corticosteroids was also specified.

### Software

Data set preparation, exploration, visualization, and analysis, including descriptive statistics, were performed using R version 3.4.3 and Comprehensive R Archive Network packages. Non-linear mixed-effect modeling using the first-order conditional estimation algorithm with interaction [Non-Linear Mixed-Effect Modeling tool (NONMEM) version 7.3; ICON Development Solutions, Ellicott City, MD, USA] [26] was used for Bayesian estimation of individual PK parameters. Logistic regression used the generalized linear model function in R with family “binomial” (variance = binomial; link = logit). Monte Carlo PK simulations were implemented using NONMEM version 7.3, and simulation data sets to assess were created using R.

## Results

### Pooled ER analysis of efficacy

**Fig. 1:**
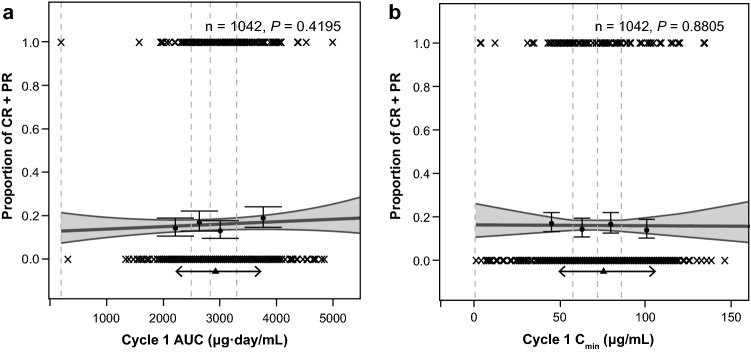
Pooled exposure–response analyses of patients with locally advanced or metastatic NSCLC or UC. The proportions of responders are plotted vs (**a**) AUC or (**b**) *C*_min_ at cycle 1. In part (**a**), for legibility, 1 extreme AUC value (> 15,000 μg.day/mL) is not displayed on the plot. Wald *P* values from logistic regression of the proportion of responders vs exposure are displayed. Gray solid lines and shaded areas represent the logistic regression slope model and 95% PI. Filled circles and error bars represent the proportions of responders in exposure quartiles and 95% CI; vertical lines are the limits of the exposure quartiles. Cross markings (×) represent response events (0: no, 1: yes). Triangle and two-headed arrows represent the mean exposure and exposure interval between the 10th and 90th percentiles, respectively, for patients receiving atezolizumab 1200 mg q3w. Cycle 1 AUC corresponds to the AUC during the first 3 weeks after treatment start and with PK parameters estimated based on cycle 1 data only. *AUC* area under the concentration–time curve, *C*_*min*_ minimum (trough) serum atezolizumab concentration, *CR* complete response, *n* number of patients, *NSCLC* non-small cell lung cancer, *PI* prediction interval*, PK* pharmacokinetics, *PR* partial response, *UC* urothelial carcinoma

Online resource Table S1 summarizes the patient populations and dosing regimens for the analyses in this study. A total of 164 of 1042 evaluable patients with exposure data (15.7%) achieved a confirmed investigator-assessed ORR. No difference in ORR was observed between patients with NSCLC (15.6%, *n* = 501) or UC (15.9%, *n* = 541), so tumor type was not included in the logistic regression models. Neither AUC nor Cmin at cycle 1 was significantly related to the probability of response (Fig. 1).

**Fig. 2:**
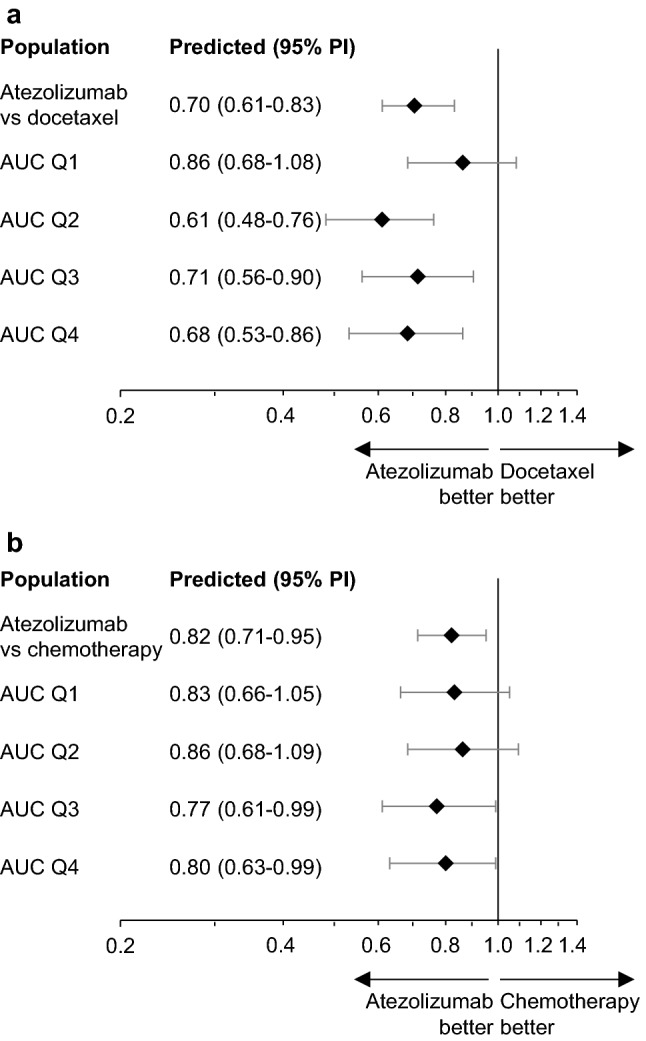
Predicted OS HRs (atezolizumab vs comparator) by cycle 1 AUC quartiles for patients with median baseline covariates. Forest plots for OS HRs from (**a**) OAK (NSCLC) and (**b**) IMvigor211 (UC) are shown. Model-predicted HRs are shown as diamonds, with bars indicating 95% PIs (1000 replicates). *AUC* area under the concentration–time curve, *HR* hazard ratio, *NSCLC* non-small cell lung cancer, *OS* overall survival, *PI* prediction interval, *UC* urothelial carcinoma

For OS, a multivariate model was developed to account for baseline prognostic factors and TGI metrics. Median OS in OAK patients with NSCLC [*n* = 388 TGI evaluable of 425 intention-to-treat (ITT) patients (91%)] was 467 days (95% CI 402–508 days) and in IMvigor211 patients with UC [*n* = 382 TGI evaluable of 467 ITT patients (82%)] was 344 days (95% CI 290–383 days). Based on this difference, tumor type was incorporated in the model. Of 770 TGI-evaluable patients, 764 had exposure data. Individual estimates of Log(tumor growth rate [KG]) and baseline factors (e.g., Eastern Cooperative Oncology Group performance status > 0; tumor size; albumin, lactate dehydrogenase, and alkaline phosphatase levels; PD-L1 status; and tumor type) were independent predictors of OS (Table S2). Of note, after accounting for baseline covariates in the final model, cycle 1 atezolizumab exposure (AUC, *C*_min_, or *C*_max_) was not significant (*P* > 0.01). The model performed well in simulating OS distribution and HRs by exposure quartiles for each tumor type even if exposure was not in the model. Comparisons of predicted and observed OS data are included in online resource Fig. S1 and Fig. S2. The flat ER relationship of atezolizumab was also illustrated in a simulation of the HRs by AUC quartiles after adjusting for baseline covariates (fixed to median values) and resampling KG as estimated (Fig. 2).

### Pooled ER analysis of safety

**Fig. 3:**
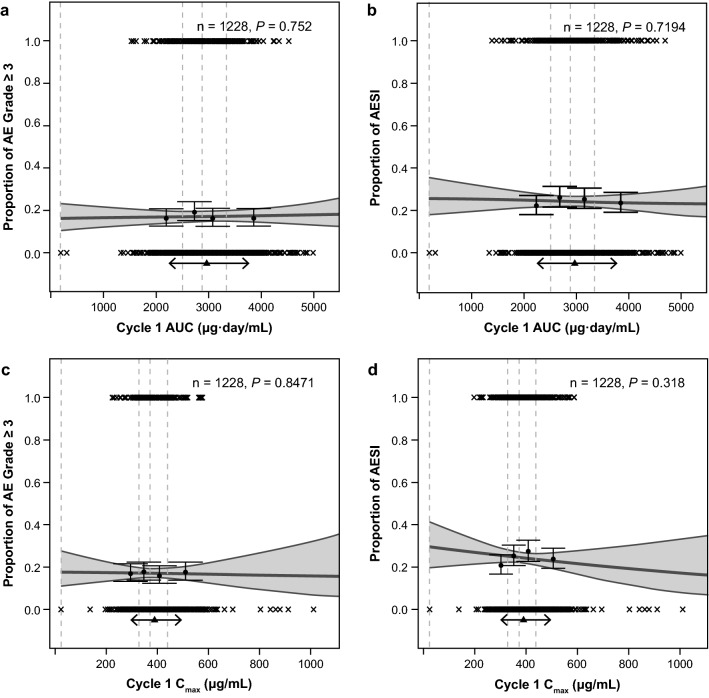
Pooled exposure–response analyses of safety in patients with locally advanced or metastatic NSCLC or UC. Indicated AE frequencies ([**a**, **c**] grade ≥ 3 AEs; [**b**, **d**] AESIs) are plotted vs (**a**, **b**) AUC or (**c**, **d**) *C*_max_ at cycle 1. For legibility, 2 extreme AUC values (> 15,000 μg.day/mL) and 2 extreme *C*_max_ values (> 1500 μg/mL) are not displayed on the plots. Wald *P* values from logistic regression of AE incidence vs exposure are displayed. Gray solid lines and shaded areas represent the logistic regression slope model and 95% PI. Filled circles and error bars represent AE proportion in exposure quartiles and 95% CI; vertical lines are the limits of the exposure quartiles. Cross markings (×) represent AE events (0: no, 1: yes). Triangle and two-headed arrows represent the mean exposure and exposure interval between the 10th and 90th percentiles, respectively, for patients receiving atezolizumab 1200 mg. Cycle 1 AUC corresponds to the AUC during the first 3 weeks after treatment start and with PK parameters estimated based on cycle 1 data only. *AE* adverse event, *AESI* adverse event of special interest, *AUC* area under the concentration–time curve, *C*_*max*_ maximum serum atezolizumab concentration, *n* number of patients, *NSCLC* non-small cell lung cancer, *PI* prediction interval*, PK* pharmacokinetics, *UC* urothelial carcinoma

Pooled atezolizumab exposure–safety analyses were performed on all atezolizumab-treated patients with locally advanced or metastatic NSCLC or UC with exposure data (*n* = 1228). AEs of grade ≥ 3 and AESIs occurred in 209 (17.0%) and 298 (24.3%) of 1228 patients, respectively. AE frequencies were similar in patients with NSCLC compared with UC (14.9% vs 19.6% for grade ≥ 3 AEs; 24.6% vs 23.9% for AESIs); therefore, tumor type was not included in the logistic regression models. Neither safety analysis (incidence of grade ≥ 3 AEs or AESIs) showed any statistically significant ER relationship with the cycle 1 exposure metrics, AUC and *C*_max_ (Fig. 3).

### PopPK prediction of atezolizumab exposure for various dosing regimens

**Fig. 4:**
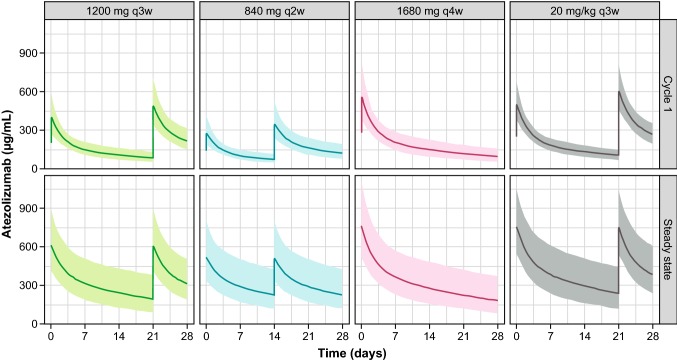
Simulated atezolizumab exposure profiles for various dosing regimens. Geometric means are plotted. Shaded areas represent 90% PIs. *PI* prediction interval, *q2w* every 2 weeks, *q3w* every 3 weeks, *q4w* every 4 weeks

Simulated atezolizumab exposure profiles for four dosing regimens—1200 mg q3w, 840 mg q2w, 1680 mg q4w, and 20 mg/kg q3w—are presented in Fig. 4. A summary of the exposure metrics associated with each dosing regimen is shown in Table 1.

**Table 1:** Summary statistics for atezolizumab exposure simulated for various regimens

Regimen	*C*_max_ (90% PI), μg/mL	*C*_min_ (90% PI), μg/mL	Weekly AUC (90% PI), μg·day/mL^a^
1200 mg q3w			
Cycle 1	403 (274–581)	85 (55–133)	1048 (763–1471)
Steady state	610 (414–891)	194 (89–383)	2115 (1264–3507)
840 mg q2w			
Cycle 1	281 (187–420)	74 (48–116)	860 (617–1237)
Steady state	517 (334–801)	226 (118–426)	2188 (1336–3733)
1680 mg q4w			
Cycle 1	563 (379–822)	97 (58–159)	1288 (887–1845)
Steady state	759 (514–1106)	182 (87–369)	2217 (1357–3705)
20 mg/kg q3w			
Cycle 1	501 (378–665)	107 (70–149)	1305 (1002–1683)
Steady state	753 (544–1038)	238 (115–443)	2596 (1592–4140)

Geometric means with 90% PIs (for 500 patients) are shown

*AUC* area under the concentration–time curve, *C*_*max*_ maximum serum atezolizumab concentration, *C*_*min*_ minimum (trough) serum atezolizumab concentration, *PI* prediction interval, *q2w* every 2 weeks, *q3w* every 3 weeks, *q4w* every 4 weeks

^a^Weekly AUC over 3 weeks (for q3w regimens), over 4 weeks (for q4w regimen), and over 2 weeks (for q2w regimen)

For the 840 mg q2w dosing regimen, the predicted *C*_min_ was 13% lower at cycle 1 and 16% higher at steady state than that for the 1200 mg q3w dosing regimen and was also in excess of the *C*_min_ target concentration of 6 μg/mL [7] by > tenfold. The 840 mg q2w predicted *C*_max_ was lower than the 1200 mg q3w *C*_max_ at cycle 1 and steady state. For the 1680 mg q4w dosing regimen, the predicted *C*_min_ was 14% higher at cycle 1 and 6% lower at steady state than that for the 1200 mg q3w dosing regimen and was also in excess of the *C*_min_ target concentration of 6 μg/mL by > tenfold. The 1680 mg q4w-predicted *C*_max_ was 12% higher at cycle 1 and 0.8% higher at steady state relative to the predicted geometric mean *C*_max_ for the 20 mg/kg dosing regimen, and was consistent with observed exposures for the 20 mg/kg q3w dosing regimen in PCD4989g [6, 27]. The predicted weekly AUC for the regimens of 840 mg q2w and 1680 mg q4w at steady state were higher than those simulated for 1200 mg q3w by 3.5% and 4.8%, respectively.

When considering fixed-dose regimens, since clearance and volume are impacted by body weight in the atezolizumab popPK model [6], patients with lower body weight would be expected to exhibit higher atezolizumab exposure relative to heavier patients. To further evaluate the q2w and q4w regimens, *C*_min_ or *C*_max_ was simulated by quartiles of body weight for dose levels of 840 mg q2w and 1680 mg q4w (Table S3). For the 1680 mg q4w regimen, the predicted *C*_max_ values for the lowest body weight quartile (< 63.7 kg, with a majority of females) were 692 and 950 μg/mL for cycle 1 and steady state, respectively, which is within the range of the observed *C*_max_ values for 1200 mg q3w and 20 mg/kg q3w [6, 27]. For the 840 mg q2w regimen, the predicted *C*_min_ values for the highest body weight quartile (> 90.9 kg, with a majority of males) were 58 and 158 μg/mL for cycle 1 and steady state, respectively, which is within the range of the observed *C*_min_ values for 1200 mg q3w and above the *C*_min_ target concentration of 6 μg/mL.

### Validation of popPK-predicted 840 mg q2w exposure

**Fig. 5:**
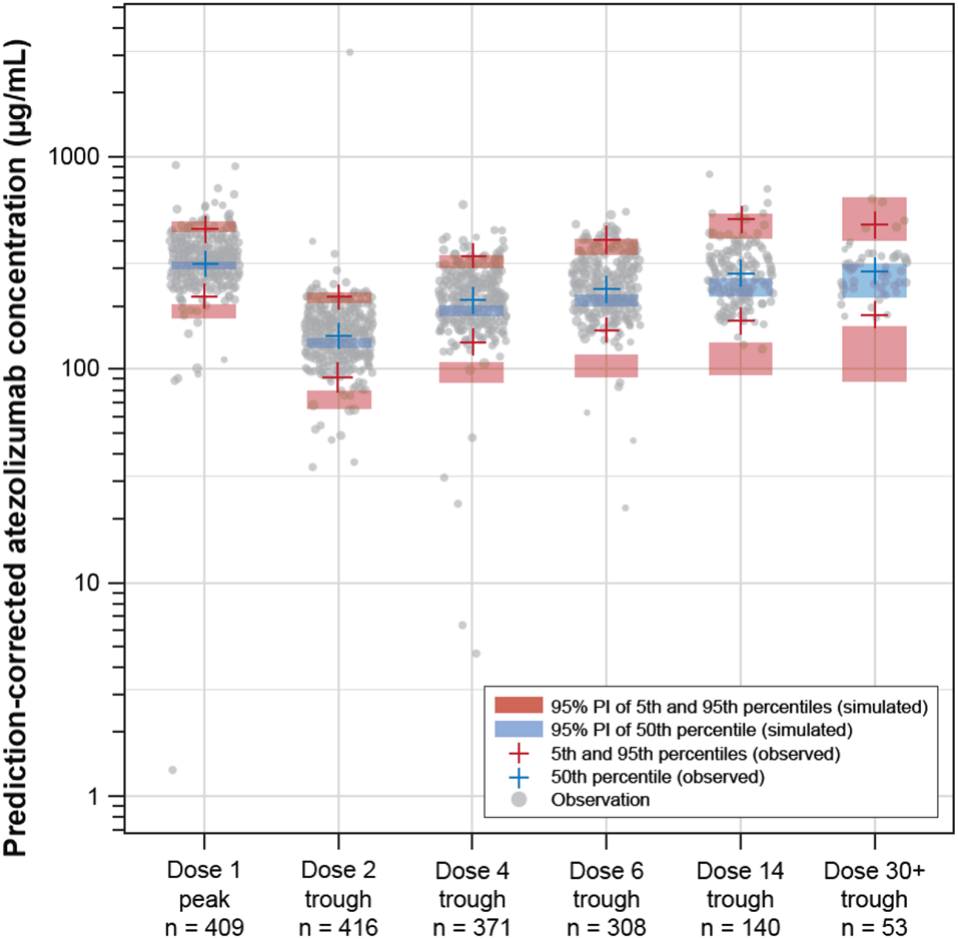
Prediction-corrected VPC of atezolizumab data in TNBC (IMpassion130) using the phase 1 popPK model. Data are plotted on a semi-log scale. Two population-predicted concentrations < 1 μg/mL are not displayed on this plot. *n* number of samples, *PI* prediction interval, *popPK* population pharmacokinetics, *TNBC* triple-negative breast cancer, *VPC* visual performance check

As an external evaluation of phase 1 popPK model and to confirm the 840 mg q2w PK simulations, the PK of the atezolizumab plus nab-paclitaxel q2w arm from the IMpassion130 study were simulated based on baseline patient covariates (pcVPC). Four-hundred forty-three (of 445) atezolizumab-treated patients had evaluable serum samples for PK analysis, for a total of 2232 samples. Results are presented in Fig. 5. Both dose 1 and steady-state exposure metrics were similar to those predicted for the 840 mg q2w dosing regimen based on the phase 1 popPK model. A trend toward underprediction of the median and fifth percentile of atezolizumab exposure data (troughs) after longer-term administration (doses 2, 4, 6, 14, and 30 +) was observed for the popPK model, consistent with the time-dependent clearance of atezolizumab [4].

### Safety by *C*_max_ relative to that predicted for 1680 mg q4w

Table S4 provides a safety summary for 20 mg/kg q3w atezolizumab-treated patients in PCD4989g, with observed *C*_max_ during cycle 1 relative to the mean predicted *C*_max_ of the 1680 mg q4w regimen. In general, AE frequencies were similar between these groups. Similar results were obtained in groups based on the PCD4989g patients’ modeled *C*_max_ (i.e., individual predictions estimated by the popPK model) relative to the mean predicted *C*_max_ of the 1680 mg q4w regimen.

### Safety by dose group

Table S5 provides a summary of atezolizumab exposure by dose group. In a dose range from 10 mg/kg q3w to 20 mg/kg q3w and 1200 mg q3w, the median treatment duration ranged from 2.07 to 9.48 months, and the median number of doses ranged from 4 to 14.5. Table S6 provides a safety summary for PCD4989g patients by dose group. The overall safety profile was consistent among the 15 mg/kg q3w, 20 mg/kg q3w, and 1200 mg q3w dose groups. Patients in the 10 mg/kg q3w dose group demonstrated increased frequency of serious AEs and treatment-related AEs relative to the other dose groups. This may be due to the longer safety follow-up and the lower number of patients in this dose group relative to the other dose groups.

### Safety by body weight

Table S7 provides a safety summary for PCD4989g and OAK patients by body weight. Median body weight in the 20 mg/kg treatment group in PCD4989g was 78.2 kg (Q1–Q3, 63.7–93.0 kg), and the overall safety profile was generally similar between patients in the lowest (*n* = 37) and upper 3 (*n* = 109) body weight quartiles. A higher incidence of grade 3–5 AEs (48.7% vs 37.3%) in the lowest body weight quartile subgroup was observed, which was due to grade 3 AEs (38.8% vs 27.8%). Evaluation of grade 3 AEs did not identify any individual AE preferred term with a  ≥ 2% difference between subgroups. Serious AEs with a  ≥ 5% difference between subgroups included fatigue and asthenia (both common to malignancy) as well as pneumonia and cardiac tamponade (known complications of thoracic cancers), with all such events occurring infrequently. In the lowest body weight subgroup, only asthenia and respiratory complications led to study treatment withdrawal; no action with respect to study treatment was taken for the other events. To assess the impact of body weight in a larger cohort of patients, AE data from OAK (1200 mg q3w dosing) were also analyzed. Median body weight was 71.0 kg (Q1–Q3, 59.5–82.2 kg). No differences between the lowest (*n* = 152) and upper 3 (*n* = 442) body weight quartiles were observed.

## Discussion

Results from this study support the interchangeable use of 840 mg q2w, 1200 mg q3w, and 1680 mg q4w dosing regimens for atezolizumab, as they are anticipated to demonstrate comparable efficacy and safety profiles while offering patients greater flexibility and convenience in their treatment. Briefly, data from all evaluated dose levels using a q3w dosing frequency, including 1200 mg q3w and 20 mg/kg q3w (the MAD in the phase 1 study PCD4989g), demonstrated that there was not a clinically meaningful exposure–efficacy or exposure–safety relationship. These data suggest that if a new dosing regimen achieves an exposure within the observed exposure range for 1200 mg q3w or 20 mg/kg q3w, it is not likely to impact efficacy or safety. PK simulations suggested that the new dosing regimens, 840 mg q2w and 1680 mg q4w, are predicted to achieve generally comparable exposure to that of the currently approved regimen of 1200 mg q3w and are within the range of observed exposures from the 1200 mg q3w and 20 mg/kg dose levels. Further characterization of the observed safety profile of patients with a *C*_max_ above and below the predicted *C*_max_ of the 1680 mg q4w regimen also supports that the safety profile of 1680 mg q4w is anticipated to be similar to the clinical experience with the q3w regimen.

Several studies of atezolizumab combination regimens have implemented 840 mg q2w dosing to be more compatible with the dosing schedule of combination agents, including the phase 3 study IMpassion130 [16]. Our popPK model simulations of an 840 mg q2w regimen indicated that this dosing achieved comparable overall exposure to that of the 1200 mg q3w regimen; the *C*_max_ and *C*_min_ values were within the observed 1200 mg q3w range, with the predicted *C*_min_ exceeding the target concentration of 6 µg/mL by at least tenfold. When applying the phase 1 popPK model to simulate IMpassion130 data, the pcVPC suggested adequate model performance for the median cycle 1 *C*_max_. A trend toward underprediction of the median and fifth percentile of atezolizumab exposure was observed after long-term treatment, consistent with the aforementioned observations of time-dependent decreases in atezolizumab clearance over time [4].

The PK simulations of a 1680 mg q4w dosing regimen (equivalent to a 21 mg/kg q3w dosing regimen for an 80-kg patient) also indicated comparable overall exposure to the currently approved regimen of 1200 mg q3w, while the predicted steady-state *C*_min_ was 6% lower than that for the currently approved regimen; this concentration also exceeded the target concentration. A small increase in cycle 1 and steady-state geometric mean *C*_max_ (12% and 0.8%, respectively) was anticipated when compared with the 20 mg/kg dose; however, the predicted *C*_max_ for the 1680 mg q4w regimen was within the range observed in the phase 1 study PCD4989g. Further, patients from PCD4989g treated at 20 mg/kg q3w had comparable safety regardless of whether their *C*_max_ was above or below the predicted cycle 1 values for the 1680 mg q4w regimen.

Similar to observations with the 1200 mg q3w regimen [6], the impact of body weight on exposure is not anticipated to be clinically meaningful for the 840 mg q2w or 1680 mg q4w regimens, as the predicted exposures for patients with low and high body weight are within range of observed exposures from the 1200 mg q3w and 20 mg/kg dose levels. These results are also further supported by a safety analysis from studies PCD4989g and OAK by body weight, which demonstrated that the overall observed safety profile was generally similar between patients in the lowest and upper 3 body weight quartiles.

The maintenance of consistent *C*_min_ levels of a protein therapeutic is considered to not only provide the most consistent disease control but also to minimize the likelihood of development of ADAs. Clinical data from TNF inhibitor studies show that episodic exposure to a protein therapeutic (i.e., exposure followed by complete washout, followed by re-exposure) is more likely to induce an immune response than the consistent presence of the same protein at the same level [28, 29]. The predicted *C*_min_ levels of the 840 mg q2w and 1680 mg q4w regimens are well in excess of the target concentration (6 μg/mL) and are within range of *C*_min_ values of the approved 1200 mg q3w regimen. Therefore, it is not anticipated that the 840 mg q2w or 1680 mg q4w regimens would result in a complete washout and re-exposure cycle that would lead to a higher immunogenicity rate than the approved 1200 mg q3w regimen.

The ability to administer atezolizumab at a less frequent dosing regimen (i.e., 1680 mg q4w) provides patients, caregivers, and healthcare providers greater flexibility and convenience. As atezolizumab is administered intravenously, the 1680 mg q4w dosing regimen is likely to reduce the time needed to receive treatment (e.g., number of visits to treatment centers) relative to a regimen dosed more frequently. In addition, the ability to switch regimens throughout treatment will also allow for greater flexibility as the dosing schedule can be matched to meet the evolving needs of each individual patient. Related approaches allowing patients and providers the option to choose between several dosing frequencies have also been applied to other checkpoint inhibitors indicated in some tumor types [8, 30–34].

Atezolizumab regimens of 840 mg q2w and 1680 mg q4w are expected to have comparable efficacy and safety to the approved regimen of 1200 mg q3w, given that the predicted exposures are within the range of observed exposures and there is no clinically meaningful ER relationship. Further, as atezolizumab PKs are consistent between indications and in combination with various agents evaluated (including, but not limited to, chemotherapy, antineoplastic drugs, and tyrosine kinase inhibitors), these results are applicable across indications where atezolizumab is administered either as monotherapy or in combination. This modeling and simulation approach, whereby new dosing regimens are identified through “exposure matching” and supported by the ER relationship and observed safety across dose levels, is in line with the model-informed drug development pilot programs endorsed by the FDA [8, 9]. In summary, this analysis supports the interchangeable use of atezolizumab dosing regimens of 840 mg q2w, 1200 mg q3w, and 1680 mg q4w, offering patients greater flexibility and convenience during their atezolizumab treatment. These data contributed to the expansion of atezolizumab dosing regimens for certain types of cancers by the FDA [4].

## Electronic supplementary material

Below is the link to the electronic supplementary material.

Supplementary material 1 (EPS 2224 kb)

Supplementary material 2 (PPTX 67 kb)

Supplementary material 3 (DOCX 30 kb)
