# Supplementary material for: Alternative dosing regimens for atezolizumab: an example of model-informed drug development in the postmarketing setting
Source: Cancer Chemother Pharmacol. 2019 Sep 21;84(6):1257–67. doi: 10.1007/s00280-019-03954-8 (PMC6820606; doi:10.1007/s00280-019-03954-8)
Supplement: Supplementary file 2 — Supplementary material 2 (PPTX 67 kb) [file 280_2019_3954_MOESM2_ESM.pptx]

## Slide 1
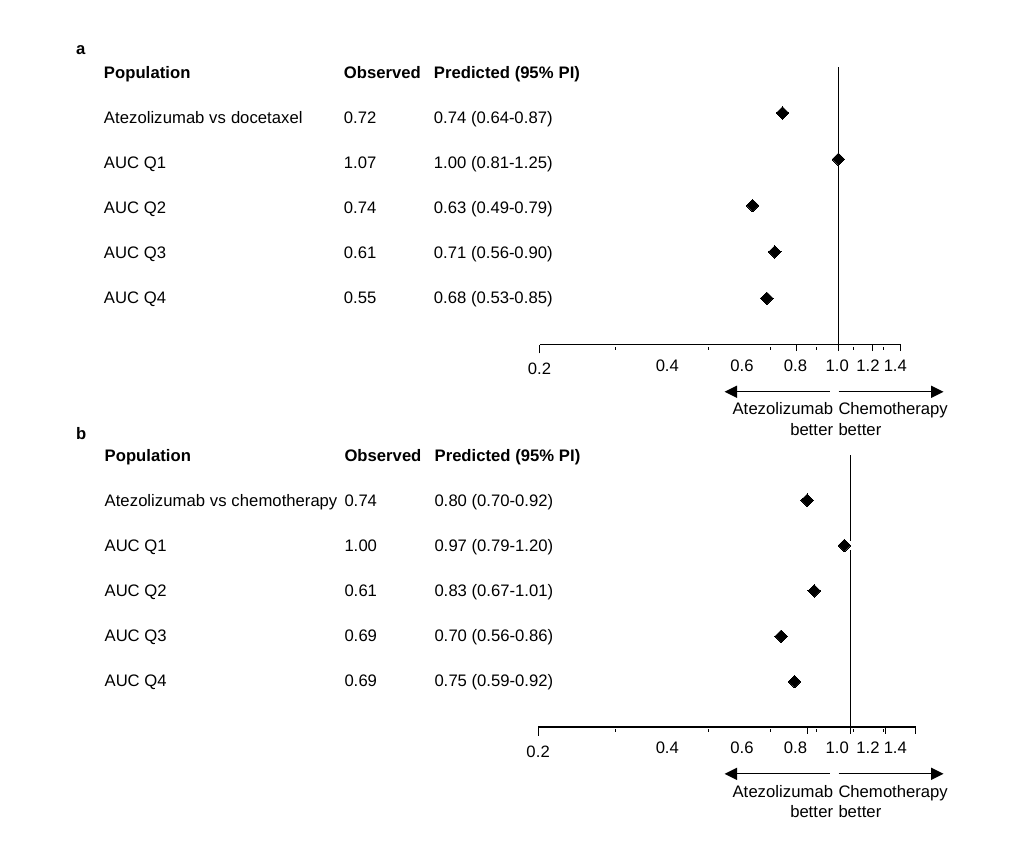

a
| Population | Observed | Predicted (95% PI) |
| --- | --- | --- |
| Atezolizumab vs docetaxel | 0.72 | 0.74 (0.64-0.87) |
| AUC Q1 | 1.07 | 1.00 (0.81-1.25) |
| AUC Q2 | 0.74 | 0.63 (0.49-0.79) |
| AUC Q3 | 0.61 | 0.71 (0.56-0.90) |
| AUC Q4 | 0.55 | 0.68 (0.53-0.85) |
### Chart
| Category | Predicted HRs | Observed |
|---|---|---|0.4
0.6
0.8
1.0
1.2
1.4
Atezolizumab
better
Chemotherapy
better
b
| Population | Observed | Predicted (95% PI) |
| --- | --- | --- |
| Atezolizumab vs chemotherapy | 0.74 | 0.80 (0.70-0.92) |
| AUC Q1 | 1.00 | 0.97 (0.79-1.20) |
| AUC Q2 | 0.61 | 0.83 (0.67-1.01) |
| AUC Q3 | 0.69 | 0.70 (0.56-0.86) |
| AUC Q4 | 0.69 | 0.75 (0.59-0.92) |
### Chart
| Category | Predicted HRs | Observed |
|---|---|---|0.4
0.6
0.8
1.0
1.2
1.4
Atezolizumab
better
Chemotherapy
better
