# Supplementary material for: Alternative dosing regimens for atezolizumab: an example of model-informed drug development in the postmarketing setting
Source: Cancer Chemother Pharmacol. 2019 Sep 21;84(6):1257–67. doi: 10.1007/s00280-019-03954-8 (PMC6820606; doi:10.1007/s00280-019-03954-8)
Supplement: Supplementary file 3 — Supplementary material 3 (DOCX 30 kb) [file 280_2019_3954_MOESM3_ESM.docx]

# Supplementary material: Online resource

**Article title:** Alternative dosing regimens for atezolizumab: an example of model-informed drug development in the postmarketing setting

**Journal:** *Cancer Chemotherapy and Pharmacology*

**Authors:** Morrissey KM, Marchand M, Patel H, Zhang R, Wu B, Chan HP, Mecke A, Girish S, Jin JY, Winter HR, Bruno R

**Corresponding author:** Kari M. Morrissey**,** Clinical Pharmacology, Genentech, Inc, 1 DNA Way, South San Francisco, CA 94080, USA. Email: morrissey.kari@gene.com

Fig. S1 Validation of TGI-OS model in simulating OS distributions by AUC (cycle 1, µg.day/mL) quartiles. Observed Kaplan-Meier OS distributions with censored data (+ symbol) from (a) OAK (NSCLC) and (b) IMvigor211 (UC) are plotted. Shaded areas represent 95% PIs for OS distributions. For interval notation format [a, b), a is included and b is excluded, such that a ≤ x < b. *AUC* area under the concentration-time curve (0 to 21 days), *NSCLC* non-small cell lung cancer, *OS* overall survival, *PI* prediction interval, *TGI* tumor growth inhibition, *UC* urothelial carcinoma

**Fig. S2** Validation of TGI-OS model in simulating HRs (atezolizumab vs comparator) by cycle 1 AUC quartiles for patients with original covariates. Forest plots for OS HRs from **(a)** OAK (NSCLC) and **(b)** IMvigor211 (UC) are shown. Observed HRs are shown as squares, and model-predicted HRs are shown as diamonds, with bars indicating 95% PIs (1000 replicates). *Atezo* atezolizumab, *AUC* area under the concentration-time curve, *Chemo* chemotherapy, *Cmin* minimum (trough) serum atezolizumab concentration, *Doce* docetaxel, *HR* hazard ratio, *NSCLC* non-small cell lung cancer, overall survival, *PI* prediction interval, *TGI* tumor growth inhibition, *UC* urothelial carcinoma

Table S1 Studies included in analysis

| Study | PCD4989g | PCD4989g | OAK | IMvigor211 | IMpassion130 |
| --- | --- | --- | --- | --- | --- |
| Population^a^ | NSCLC cohort | UC cohort | NSCLC | UC | Previously untreated locally advanced or metastatic TNBC |
| Clinical phase | 1 | 1 | 3 | 3 | 3 |
| Patients, n^b^ | 88 | 92 | Atezolizumab arm  422^c,d^  613^e^  Chemotherapy arm  401^d^  612^e^ | 467 (atezolizumab arm)  464 (chemotherapy arm) | 451 (atezolizumab + nab-paclitaxel arm)  451 (placebo + nab-paclitaxel arm) |
| Atezolizumab-exposed patients, n^f^ | 87 | 90 | 414^d^  596^e^ | 455 | 443 (atezolizumab + nab-paclitaxel arm) |
| Atezolizumab dose | IV q3w  1 mg/kg (n = 1)  10 mg/kg (n = 10)  15 mg/kg (n = 27)  20 mg/kg (n = 49) | IV q3w  15 mg/kg (n = 84)  20 mg/kg (n = 1)^g^  1200 mg (n = 5) | IV q3w  1200 mg | IV q3w  1200 mg | IV q2w  840 mg (in combination with nab-paclitaxel) |
| Patients for exposure-response analyses, n  Exposure-efficacy (ORR)  TGI-OS modeling  Exposure-safety | 87  -  87 | 90  -  90 | 414  388  596 | 451  382  455 | -  -  - |

*ITT* intention to treat, *IV* intravenous, *n* number of patients, *NSCLC* non-small cell lung cancer, *ORR*, objective response rate, *PK* pharmacokinetics, *q2w* every 2 weeks, *q3w* every 3 weeks, *TGI-OS* tumor growth inhibition-overall survival, *TNBC* triple-negative breast cancer, UC urothelial carcinoma

^a^ Cohorts from PCD4989g and patients in OAK and IMvigor211 had locally advanced or metastatic disease. Patients in OAK and IMvigor211 had progression during or following platinum-containing chemotherapy

^b^ Refers to enrolled or ITT populations

^c^ Twenty-seven of the first 850 patients did not receive treatment

^d^ First 850 patients enrolled

^e^ All 1225 patients enrolled

^f^ Refers to patients who received ≥ 1 dose and for whom ≥ 1 evaluable PK sample was obtained

^g^ Patient’s dose was incorrectly recorded as 20 mg/kg but was actually 15 mg/kg, which was used for deriving exposure

Table S2 Parameter estimates of final multivariate OS model in OAK and IMvigor211 with mUC tumor type

as a factor

| Parameter | Estimate | SE | Z | *P* |
| --- | --- | --- | --- | --- |
| Intercept | 2.946 | 0.3142 | 9.377 | 6.776e-21 |
| mUC tumor type | -0.1661 | 0.06302 | -2.636 | 0.008378 |
| Log(KG, week^-1^) | -0.6185 | 0.03816 | -16.21 | 4.372e-59 |
| ECOG PS > 0 | -0.3406 | 0.06253 | -5.447 | 5.13e-08 |
| Albumin (g/L) | 0.02767 | 0.006164 | 4.489 | 7.169e-06 |
| Tumor burden (mm) | -0.002817 | 0.0006747 | -4.175 | 2.974e-05 |
| Lactate dehydrogenase (IU) | -0.0005352 | 0.0001895 | -2.824 | 0.004739 |
| IC2/3 (vs IC0/1) | 0.2535 | 0.08514 | 2.977 | 0.002908 |
| Alkaline phosphatase (IU) | -0.001383 | 0.0003242 | -4.265 | 1.998e-05 |
| Log(scale) | -0.3191 | 0.03497 | -9.125 | 7.183e-20 |

Survival time was analyzed in days

*ECOG PS* Eastern Cooperative Oncology Group performance status, *IC* PD-L1 expression on tumor-infiltrating immune cells, *KG* tumor growth rate constant from tumor growth inhibition model, *mUC* metastatic urothelial carcinoma, *OS* overall survival, *P* Wald test *P* value, *Scale* standard deviation of log(OS), *SE* standard error of parameter estimate, *Z* Wald test statistic

Table S3 Simulated atezolizumab Cmax and Cmin values by body weight quartile

|  |  | Body weight quartile, kg^a^ | | | |
| --- | --- | --- | --- | --- | --- |
|  |  | [36.5, 63.7) | [63.7, 77.0) | [77.0, 90.9) | [90.9, 168.0] |
| 840 mg q2w | Cmin (90% PI), μg/mL |  |  |  |  |
|  | Cycle 1 | 93 (64-136) | 77 (54-110) | 67 (45-98) | 58 (40-84) |
|  | Steady state | 299 (165-549) | 241 (132-426) | 197 (103-366) | 158 (78-296) |
| 1680 mg q4w | Cmax (90% PI), μg/mL |  |  |  |  |
|  | Cycle 1 | 692 (505-950) | 573 (407-784) | 506 (368-675) | 425 (313-586) |
|  | Steady state | 950 (692-1325) | 781 (564; 1052) | 683 (499-939) | 562 (405-777) |

Geometric means with 90% PIs (for 500 patients) are shown.

*Cmax* maximum serum atezolizumab concentration, *Cmin* minimum (trough) serum atezolizumab concentration, *PI* prediction interval*, q2w* every 2 weeks, *q4w* every 4 weeks

^a^ For interval notation format [a, b), a is included, and b is excluded, such that a ≤ x < b

Table S4 AE summary by observed or modeled Cmax during cycle 1: phase 1 PCD4989g patients receiving atezolizumab 20 mg/kg q3w

| Patients with ≥ 1 indicated AE, n (%) | Patients with indicated Cmax relative to predicted mean Cmax for 1680 mg q4w during cycle 1 | | | |
| --- | --- | --- | --- | --- |
|  | Observed ≤ predicted (n = 98) | Observed > predicted  (n = 40) | Modeled ≤ predicted  (n = 117) | Modeled > predicted  (n = 28) |
| Any AE | 97 (99.0) | 40 (100.0) | 116 (99.1) | 28 (100.0) |
| Total deaths | 70 (71.4) | 24 (60.0) | 81 (69.2) | 19 (67.9) |
| AE with fatal outcome | 2 (2.0) | 0 | 2 (1.7) | 0 |
| Serious AE | 43 (43.9) | 15 (37.5) | 49 (41.9) | 12 (42.9) |
| Grade 3-5 AE | 52 (53.1) | 14 (35.0) | 61 (52.1) | 10 (35.7) |
| AE leading to treatment withdrawal | 5 (5.1) | 1 (2.5) | 7 (6.0) | 0 |
| AESI | 47 (48.0) | 18 (45.0) | 54 (46.2) | 15 (53.6) |
| AESI requiring corticosteroids | 8 (8.2) | 4 (10.0) | 12 (10.3) | 2 (7.1) |
| AE within 24 hours of infusion | 78 (79.6) | 38 (95.0) | 96 (82.1) | 26 (92.9) |

Atezolizumab-treated safety-evaluable patients were included

*AE* adverse event, *AESI* adverse event of special interest, *Cmax* maximum serum atezolizumab concentration, *q3w* every 3 weeks, *q4w* every 4 weeks

**Table S5** Atezolizumab exposure by dose group: atezolizumab-treated patients from PCD4989g

|  | 10 mg/kg | 15 mg/kg | 20 mg/kg | 1200 mg |
| --- | --- | --- | --- | --- |
|  | q3w IV | q3w IV | q3w IV | q3w IV |
|  | (n = 36) | (n = 236) | (n = 146) | (n = 228) |
| Treatment duration (months) |  |  |  |  |
| n | 36 | 236 | 146 | 228 |
| Mean (SD) | 15.38 (18.17) | 10.44 (15.93) | 8.55 (11.98) | 4.43 (7.18) |
| Median (Min-Max) | 9.48 (0.0-67.0) | 3.42 (0.0-64.7) | 4.62 (0.0-69.1) | 2.07 (0.0-40.7) |
| Number of doses |  |  |  |  |
| n | 36 | 236 | 146 | 228 |
| Mean (SD) | 16.5 (15.3) | 14.0 (19.3) | 11.5 (13.5) | 7.1 (10.1) |
| Median (Min-Max) | 14.5 (1-61) | 6 (1-79) | 7 (1-96) | 4 (1-60) |

Table S6 AE summary by dose group: atezolizumab-treated patients from PCD4989g

|  | 10 mg/kg | 15 mg/kg | 20 mg/kg | 1200 mg |
| --- | --- | --- | --- | --- |
|  | q3w IV | q3w IV | q3w IV | q3w IV |
| Patients with ≥ 1 indicated AE, n (%) | (n = 36) | (n = 236) | (n = 146) | (n = 228) |
| Any AE^1^ | 35 (97.2) | 232 (98.3) | 145 (99.3) | 225 (98.7) |
| AE with fatal outcome | 1 (2.8) | 3 (1.3) | 2 (1.4) | 7 (3.1) |
| Serious AE | 20 (55.6) | 115 (48.7) | 65 (44.5) | 103 (45.2) |
| Serious AE leading to treatment withdrawal | 2 (5.6) | 9 (3.8) | 4 (2.7) | 8 (3.5) |
| Serious AE leading to dose interruption | 7 (19.4) | 41 (17.4) | 22 (15.1) | 41 (18.0) |
| AE leading to withdrawal from treatment | 2 (5.6) | 16 (6.8) | 7 (4.8) | 10 (4.4) |
| AE leading to dose interruption | 13 (36.1) | 66 (28.0) | 33 (22.6) | 69 (30.3) |
| Related AE | 31 (86.1) | 174 (73.7) | 110 (75.3) | 141 (61.8) |
| Related AE leading to treatment withdrawal | 1 (2.8) | 11 (4.7) | 3 (2.1) | 5 (2.2) |
| Related AE leading to dose interruption | 4 (11.1) | 27 (11.4) | 17 (11.6) | 25 (11.0) |

1. Per PCD4989g protocol, all adverse events were collected after treatment initiation until 90 days following the last administration of study treatment or until study discontinuation/termination or until initiation of subsequent anti-cancer therapy, whichever occurred first. Patients were contacted at 60 and 90 days after the last dose of study treatment to determine if any new adverse events had occurred. After this period, investigators reported only serious adverse events that were felt to be related to prior study treatment.

Table S7 AE summary by body weight: atezolizumab-treated patients from PCD4989g and OAK

| Patients with ≥ 1 indicated AE, n (%) | Patients from indicated study, dosing subgroup and body weight quartile(s) | | | |
| --- | --- | --- | --- | --- |
|  | PCD4989g (20 mg/kg), lowest (n = 37) | PCD4989g (20 mg/kg), upper 3 (n = 109) | OAK (1200 mg), lowest (n = 152) | OAK (1200 mg), upper 3 (n = 442) |
| Any AE | 37 (100.0) | 108 (99.1) | 142 (93.4) | 418 (94.6) |
| Total deaths | 24 (64.9) | 77 (70.6) | 98 (64.5) | 277 (62.7) |
| AE with fatal outcome | 1 (2.7) | 1 (0.9) | 5 (3.3) | 20 (4.5) |
| Serious AE | 17 (45.9) | 45 (41.3) | 51 (33.6) | 151 (34.2) |
| Grade 3-5 AE | 21 (56.8) | 51 (46.8) | 74 (48.7) | 165 (37.3) |
| AE leading to treatment withdrawal | 3 (8.1) | 4 (3.7) | 16 (10.5) | 32 (7.2) |
| AESI | 15 (40.5) | 54 (49.5) | 45 (29.6) | 150 (33.9) |
| AESI requiring corticosteroids | 3 (8.1) | 11 (10.1) | 11 (7.2) | 44 (10.0) |
| AE within 24 hours of infusion | 32 (86.5) | 90 (82.6) | 99 (65.1) | 321 (72.6) |

Atezolizumab-treated safety-evaluable patients were included

AE adverse event, AESI adverse event of special interest
